# Smoke‐Affected Rain Fertilizes Terrestrial and Aquatic Ecosystems

**DOI:** 10.1111/gcb.71050

**Published:** 2026-08-25

**Authors:** Alexandra G. Ponette‐González, Janice Brahney, Heather A. Holmes, Bryn Spielvogel, Kathleen C. Weathers

**Affiliations:** ^1^ Natural History Museum of Utah University of Utah Salt Lake City Utah USA; ^2^ Department of City & Metropolitan Planning University of Utah Salt Lake City Utah USA; ^3^ Department of Watershed Sciences and Ecology Center Utah State University Logan Utah USA; ^4^ Department of Chemical Engineering University of Utah Salt Lake City Utah USA; ^5^ Department of Parks, Recreation, and Tourism University of Utah Salt Lake City Utah USA; ^6^ Cary Institute of Ecosystem Studies Millbrook New York USA

**Keywords:** air quality, ash, atmospheric deposition, fertilization, forest, lake, particulate matter, smoke, wildfire

## Abstract

Wildland fire smoke and ash are pervasive and a significant source of particulate matter to the atmosphere. In addition to effects on air quality, wildland smoke and ash deposit to ecosystems via dry (particulate) and wet (precipitation) deposition. Here we combine remotely sensed smoke polygon data with rainfall and weekly wet deposition data from > 250 U.S. National Atmospheric Deposition Program (NADP) sites to quantify wildland smoke influence on wet ion deposition during 2014, 2020, and 2022. Combined records of daily smoke and rain indicate that sites experienced 21 smoke‐rain days (when smoke and rainfall co‐occur), on average, in 2022 compared to 4 days in 2014 and 6 days in 2020. The number of smoke‐rain days and their spatial distribution were driven more by variation in smoke than rain occurrence. Across 16 climate regions, the Upper Midwest was a persistent hotspot for smoke‐rain days in all 3 years. Mixed effects models estimating site‐level impacts of smoke‐rain on a weekly basis showed that smoke‐rain days had significant positive effects on wet deposition of all nine ions evaluated. Orthophosphate (PO_4_^3−^, 46%), potassium (K^+^, 41%), and ammonium (NH_4_^+^, 36%) deposition were most enhanced compared to non‐smoke‐rain days. The magnitude of these effects varied by region. With each additional smoke‐rain day, weekly wet NH_4_^+^ deposition increased by 100%–220% in Alaska & the Arctic, Northwest, and West regions, whereas PO_4_^3−^ deposition increased ~100% in the Northeast. Based on modeled estimates, smoke‐rain days contributed ~20%–30% of annual wet NH_4_^+^, PO_4_^3−^, K^+^, and Ca^2+^ deposition in 2022. Further, molar N:P ratios of wet deposition were higher at 76% of NADP sites during weeks with versus without smoke‐rain. Our results demonstrate that smoke‐affected wet deposition can deliver biologically important nutrients to ecosystems, which may be leading to fire‐fertilizing of terrestrial and aquatic ecosystems.

## Graphical abstract

Smoke drives increased wet deposition of essential macronutrients that limit growth and production in terrestrial and aquatic ecosystems. Smoke increases nitrogen, phosphorus, and potassium deposition most, and alters their relative ratios. In a high smoke year, smoke‐affected rain can deliver 20%–30% of annual wet deposition for these key elements. These findings suggest that wildland smoke may fertilize some terrestrial and aquatic ecosystems and could influence their species composition.

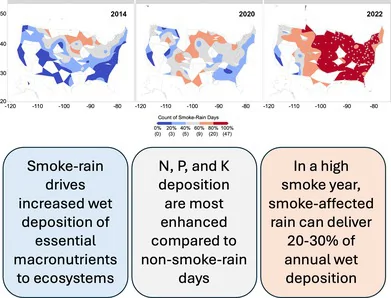

## 1 Introduction

Smoke and ash from wildland fires, which include wild and prescribed fires, are an increasingly significant component of atmospheric particulate matter (PM) (Childs et al. 2022; Xu et al. 2023). They also constitute growing environmental health threats (Paul et al. 2023; Krasovich Southworth et al. 2025). In 2020, wildfires accounted for up to 25% of annual PM_2.5_ (≤ 2.5 μm aerodynamic diameter) across the U.S. and up to 50% in some western U.S. regions (Burke et al. 2021). Population exposure to smoke PM_2.5_ is on the rise across the U.S. and globally. In the U.S., recent (i.e., 2020–2023) exposures were estimated to be as much as 6.7 times higher than in previous years (2006–2019; Childs et al. 2025). Globally, from 2010 to 2019, approximately 2 billion people experienced an average of ~10 days per year of fire‐influenced air pollution (PM_2.5_ and ozone), up 7% from the previous decade (Xu et al. 2023).

Although PM_2.5_ is of greatest concern for health impacts, wildland fires carry more and larger size classes of PM, which are subsequently deposited to ecosystems downwind (Scordo et al. 2026). The ecological impacts and risks of air pollution associated with wildland fires are less well understood than the human impacts, however (Paul et al. 2023). Emitted particles within smoke plumes can travel thousands of kilometers in the atmosphere before being deposited to the Earth's surface by dry (particulate) and wet (precipitation) deposition (Weathers and Ponette‐González 2011). In fact, ground‐based measurements and computer simulations show that smoke and ash deposition from the atmosphere constitutes a major pathway for the delivery of biologically available nutrients (e.g., ammonium and nitrate‐nitrogen, and ortho‐phosphorus) to recipient ecosystems (Brahney et al. 2015; Ponette‐González et al. 2016; Bauters et al. 2021). For example, fire emissions during the historic summer 2020 western U.S. wildfire season contributed to a 78% increase in simulated annual average total (wet + dry) nitrogen deposition in the State of California (Campbell et al. 2022). The spatiotemporal variability of smoke plumes and the heterogeneous physicochemical properties of smoke and ash (Bodí et al. 2014; Bian et al. 2020; Chandra et al. 2022; Daniels et al. 2024; Krasovich Southworth et al. 2025) suggest that wet and dry deposition influenced by wildland smoke and ash will exhibit complex chemistries as well as spatial and temporal variability.

Although the ecosystem effects of smoke‐affected deposition remain highly uncertain, recent research underscores the potential for wildland smoke and ash deposition to have important impacts on terrestrial as well as aquatic ecosystems (Koplitz et al. 2021; Farruggia et al. 2024). In forests, nitrogen deposition originating from wildland smoke can alter tree survival and growth rates, with the direction and magnitude of responses varying by species and smoke concentration (Koplitz et al. 2021; Huang et al. 2024). In aquatic ecosystems, including streams and lakes, phosphorus deposited in smoke particles (Fernandez et al. 2024) has the potential to stimulate algal production, and potentially, harmful cyanobacterial blooms (Olson et al. 2026). In marine systems, deposition of iron‐enriched smoke aerosols emitted from Australian wildfires has been linked to phytoplankton blooms in the Southern Ocean (Tang et al. 2021). In addition, smoke and ash can deliver pollutants and heavy metals to ecological systems (Kelly et al. 2021; Chandra et al. 2022; Sricharoenvech et al. 2025). In sum, smoke and ash, when deposited, can supply critical limiting nutrients and pollutants to ecosystems with both beneficial and deleterious environmental consequences.

Wildfire patterns are changing across the globe (Jones et al. 2022; Cunningham et al. 2024). Although future trajectories remain uncertain, model simulations point to geographical shifts in wildfire occurrence and intensity (Haas et al. 2026). Some climate change projections suggest that by the mid‐21st century the number of days conducive to extreme wildland fires will increase by 20%–50% (Bowman et al. 2017), fueling dramatic increases in wildland fire emissions. Better understanding how these emissions affect atmospheric deposition to downwind landscapes is critical for identifying when and where excess nutrient deposition is likely to occur as well as for predicting and managing resultant impacts.

A major challenge for researchers trying to quantify the role of wildland smoke and ash on ecosystem functioning is a lack of data. Atmospheric deposition is a fundamental pathway for nutrient and pollutant delivery to the Earth's surface (Weathers and Ponette‐González 2011), yet observational data on atmospheric deposition to ecosystems during and following fire are rare. The data that do exist are often limited in spatial extent (i.e., limited to individual sites or watersheds), temporal duration (e.g., collected over limited seasons or few years), and/or scope (e.g., data on one nutrient only, e.g., nitrogen). Observations over large spatial scales and that encompass multiple time periods are essential for comprehensive regional assessments of ecosystem vulnerability to wildland smoke and ash.

Here, we integrate observations from the ground‐based National Atmospheric Deposition Program monitoring network with remotely sensed smoke polygon data to quantify the influence of smoke‐affected rain on wet deposition of major ions. Our analysis encompasses 16 climate regions and 3 years that contrast in smoke incidence, enabling assessment of the relative effects of smoke on wet deposition across regions and ecosystems.

## 2 Materials and Methods

### 2.1 Smoke‐Rain Day Frequency and Distribution

**FIGURE 1:**
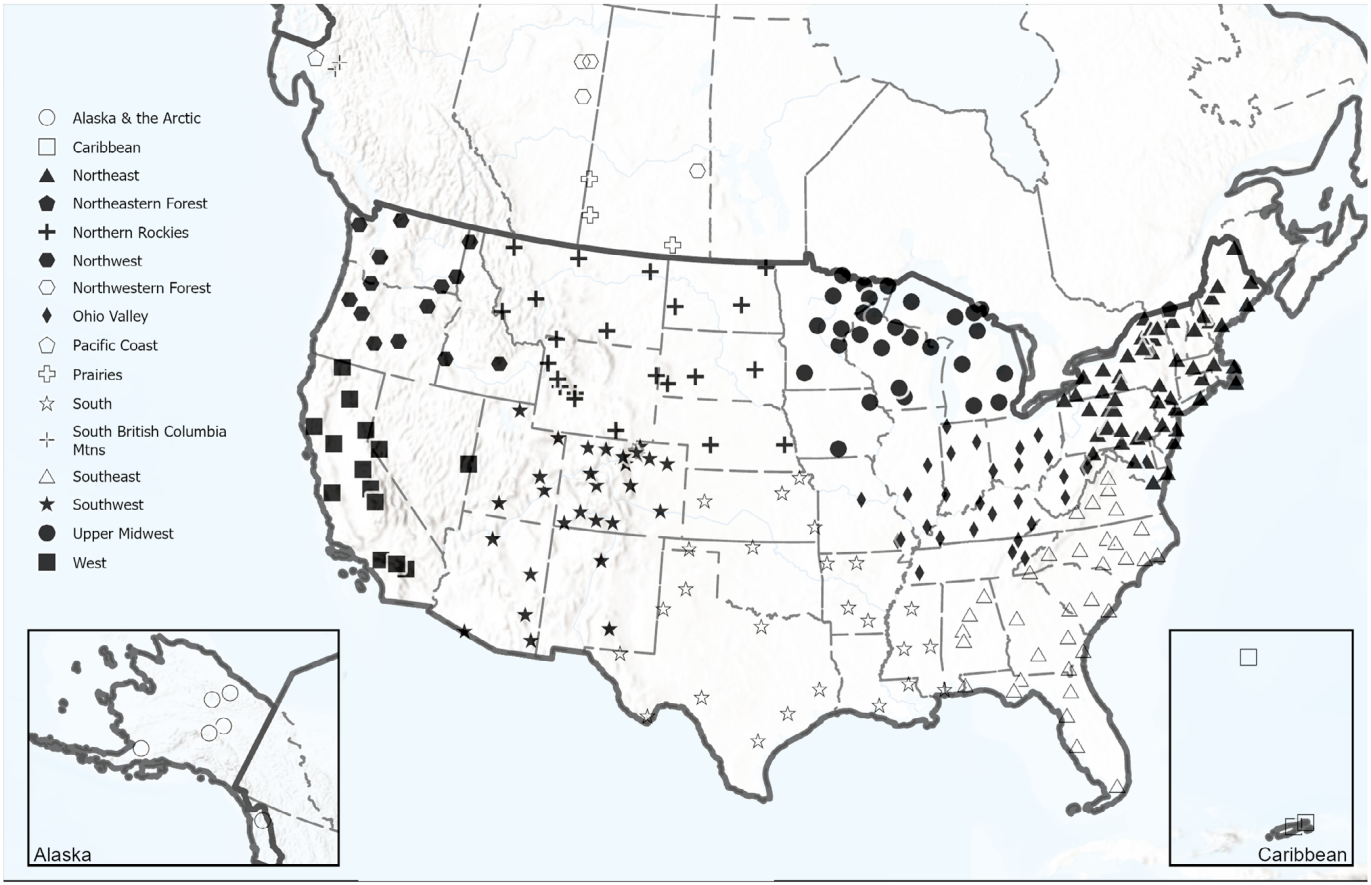
U.S. National Atmospheric Deposition Program (NADP) National Trends Network sites included in this research and grouped into 16 climate regions. Sites were active during one or all the study years: 2014, 2020, and 2022. Map lines delineate study areas and do not necessarily depict accepted national boundaries.

We examined differences in the number and spatial distribution of smoke‐rain days at U.S. National Atmospheric Deposition Program National Trends Network (hereafter NADP) monitoring sites (Figure 1). We defined smoke‐rain days as days when smoke and rainfall co‐occur. The NADP currently maintains > 250 network sites across the U.S. and Canada and their territories. The sites were grouped into 16 climate regions because rainfall, smoke, and natural and anthropogenic emissions sources vary by region (Hand et al. 2024). The regions include the Northwest, Northern Rockies, West, Southwest, South, Midwest, Ohio Valley, Southeast, and Northeast in the contiguous U.S. as delineated by Karl and Koss (1984), Alaska & the Arctic, the Caribbean, and five Canadian climate regions.

For this study, we selected three focal years—2014, 2020, and 2022—which contrast in smoke‐day frequency and smoke spatial distribution (Figure S1). Data beyond 2022 were not available when the project was initiated. The NOAA Hazard Mapping System (HMS) smoke product was used to determine the number of smoke‐affected days at each NADP site. HMS is an interactive tool developed by NOAA NESDIS that provides daily fire detections and smoke polygons for North America (https://www.ospo.noaa.gov/Products/land/hms.html; Rolph et al. 2009; Brey et al. 2018). The HMS smoke product relies on visual inspection of true color images from geostationary satellites, specifically the Geostationary Operational Environmental Satellites: GOES‐East and GOES‐West. Although GOES enables the update of smoke polygons throughout the day, we use daily HMS smoke polygons. The smoke polygons indicate smoke presence in the atmospheric column and are qualitatively classified as light, medium, or heavy density based on approximate smoke thickness, or opacity, in the satellite images. Smoke detection using visible imagery is restricted to daytime, can be limited by cloud cover (Rolph et al. 2009), in certain regions can be confused with haze (Brey et al. 2018), and can be impacted by the vertical layering of smoke in the atmosphere. However, we consider the HMS smoke product to be a reasonable smoke proxy for this study because it provides a binary smoke indicator and daily smoke days can be related to weekly wet deposition samples. In addition, wet (rainwater) deposition is less likely to be impacted by vertical layering of smoke than dry deposition given the downward transport of rain through the atmospheric column.

The HMS smoke polygons were used in conjunction with rainfall data collected at the NADP sites to identify the presence or absence of smoke‐rain days at these same sites and for each of the focal years. All NADP sites have a weighing‐bucket rain gauge that provides sub‐hourly to hourly records of rainfall amount. Rainfall is recorded to the nearest 0.254 mm. Here, we define a “smoke‐rain day” as a day when an HMS smoke plume (i.e., of any density, light, medium, or heavy smoke) is present over a NADP site and rainfall occurs at that site on the same day. A “non‐smoke‐rain day” is a day when smoke and rainfall do not occur simultaneously at a NADP site.

Although all days in our dataset had a valid HMS smoke observation, there were days when missing rainfall data precluded us from determining if a smoke‐rain day was present or absent. These cases were assigned a missing value. Daily smoke‐rain days were then summed to match the weekly NADP wet deposition data.

### 2.2 Quantifying Smoke‐Rain Effects on Wet Deposition

We examined smoke‐rain effects, meaning the effect of each additional smoke‐rain day in a week, on wet ion deposition monitored at the NADP sites. At these network sites, site operators collect wet (rainwater) deposition samples following standard operating procedures (NADP 2024). Samples are collected every Tuesday morning from an automated sampler exposed only during rainfall. Upon arrival to the Wisconsin State Laboratory of Hygiene at the University of Wisconsin‐Madison, NADP filters all rainwater samples to remove particulates. Rainwater is swirled and poured through a 47‐mm Polyethersulfone (PES) 0.45 μm Pall filter into HDPE Nalgene sample bottles. Upon request, sample filters are stored in labeled plastic petri dishes and frozen until analysis. Following filtration, rainwater samples are analyzed for: free acidity (H^+^ as pH) with a pH meter and conductance with a conductance probe within 3 days of sample receipt. Within 30 days of sample receipt at the lab, samples are analyzed for calcium (Ca^2+^), magnesium (Mg^2+^), sodium (Na^+^), and potassium (K^+^) using inductively coupled plasma‐optical emission spectroscopy; sulfate (SO_4_^2−^), nitrate (NO_3_^−^), and chloride (Cl^−^) using ion chromatography; and ammonium (NH_4_^+^) using flow injection analysis. The NADP program also analyzes orthophosphate (PO_4_^3−^) using flow injection analysis but only for quality assurance and quality control purposes, and thus these data are not made publicly available. The NADP provided PO_4_^3−^ data to the authors for this research.

In 2020, we requested sample filters for 36 rainwater samples collected at five NADP sites located in the States of California, Colorado, New Mexico, and Texas (CA96, CO15, CO92, NM07, TX16). Samples were shipped to Utah State University Environmental Biogeochemistry and Paleolimnology Lab. Charcoal particles on the sample filters were counted and measured using an Olympus BX51 microscope with digital camera and image processing software. Linear regressions of log‐transformed wet ion deposition against charcoal deposition showed significant positive relationships between ions and charcoal in wet deposition samples (Figure S2). Potassium, PO_4_^3−^, and Mg^2+^ deposition exhibited moderate relationships, whereas Ca^2+^, NH_4_^+^, and NO_3_^−^ exhibited weak relationships with charcoal on the sample filters. Relationships between SO_4_^2−^, Na^+^, and Cl^−^ and charcoal deposition were not detected. Based on these data indicating that ash can contribute to nutrient content in wet deposition, we focused on the effects of smoke‐rain days on wet deposition. We focus here on smoke given the current lack of ash monitoring and the widespread availability of remotely sensed smoke products, such as HMS, for North America.

We downloaded wet deposition data from NADP's website for each of the study years (https://nadp.slh.wisc.edu/networks/national‐trends‐network/). For all ions, concentrations at or below detection limit were set to half the minimum detection limit, and wet deposition (kg/ha) was calculated as the product of chemical concentration and rainfall amount. The wet deposition data included some samples with shorter and longer than weekly collection times. Therefore, deposition was normalized (i.e., divided) by the number of collection hours and then multiplied by 168 h (i.e., 7 days) to obtain a weekly wet deposition rate (kg/ha/week) for all samples. Similarly, the count of rain days was normalized (i.e., divided) by the number of observation days and then multiplied by 7 days to obtain count of rain days per week.

All samples considered valid by NADP quality assurance evaluations were included in our analyses. All samples deemed invalid were excluded from analyses except those that occurred during weeks with smoke‐rain days. Specifically, weekly samples assigned a code of “c” (i.e., for contamination) that contained one or more smoke‐rain days were included in the analysis given the likelihood that smoke/ash was present in the sample (Table S1). NADP excludes samples with extended sampling periods (> 194 h). For comparability among sites and weeks, we excluded wet deposition samples with limited sampling periods (< 144 h) as well, and with incomplete rainfall observations (i.e., weeks with more than 1.5 days of missing rainfall data; Table S2). The filtered data set (*n* = 24,974) was joined with the weekly smoke‐rain data.

### 2.3 Data Analysis

Spatial patterns of smoke days, rain days, and smoke‐rain days were visualized as contour plots constructed using piecewise linear interpolation of the input data based on a Delaunay triangulation. The contours show clusters of NADP sites with similar levels of smoke, rain, and smoke‐rain days.

We used mixed effects models with random intercepts to assess the influence of smoke‐rain days (i.e., number of days) within a given week on weekly wet deposition at the individual monitoring site level, accounting for site‐level variation in deposition, rainfall amount, season (i.e., categorical variables of winter = DJF, spring = MAM, summer = JJA, fall = SON), and year. Weekly wet deposition for each ion was modeled as:

$$\begin{array}{c} \log\left(\mu_{\mathrm{ij}}\right)={\text{𝛽}}_{0}+{\text{𝛽}}_{1}{\text{smokeraindays}}_{\mathrm{ij}}+{\text{𝛽}}_{2}{\text{precipitation}}_{\mathrm{ij}}\\ \;{{\text{+𝛽}}_{3}}^{⊤}\;{\text{season}}_{\text{𝑖𝑗}}+{{\text{𝛽}}_{4}}^{⊤}{\text{year}}_{\text{𝑖𝑗}+}\;u_{j} \end{array}$$

where *μ*_*ij*_ is the expected wet deposition for weekly observation *i* at site *j*; 𝛽_0_ is the intercept; 𝛽_1_ is the regression coefficient for the number of smoke‐rain days; 𝛽_2_ is the regression coefficient for weekly precipitation; 𝛽_3_ and 𝛽_4_ are vectors of coefficients representing season and year effects, respectively; and 𝑢_𝑗_ is the site‐specific random intercept.

Following the approach of Krasovich Southworth et al. (2025), we examined intra‐site changes in deposition rather than inter‐site changes because smoke occurrence varies regionally and thus inter‐site differences in wet deposition due to smoke‐affected rain could be confounded by regional variations in smoke frequency. We controlled for precipitation amount by including precipitation as a predictor in the model because at any individual monitoring site, weeks with more smoke‐rain days could also have received more rainfall, precluding our ability to isolate the effects of smoke‐rain frequency from those of rain on wet deposition. We accounted for season and year given that concentrations of major chemical species in ambient air vary seasonally and annually due to fire (Hand et al. 2024; Krasovich Southworth et al. 2025).

Because wet deposition data are highly right skewed, we first tested different models for estimating weekly wet deposition. Residual diagnostics for the standard linear mixed effects model showed violations of normality assumptions (i.e., skew and heteroscedasticity). A linear mixed effects model with natural log‐transformed deposition data improved but did not fully resolve the residual non‐normality issue. Gamma log‐link mixed effects models do not require that the assumption of residual normality be satisfied and are therefore well suited to environmental data with a high positive skew. These models showed substantially better model fit statistics across ions.

Based on diagnostic criteria and theoretical fit, we elected to use the gamma log‐link mixed effects models with site‐level random intercepts (i.e., “all sites” model). Coefficients for these models are expressed on a log scale and thus can be difficult to directly interpret. We instead report exponentiated coefficients (exp *β*) (Supporting Information), which allow for interpretation on the original outcome scale. Coefficients can be understood as multiplicative changes in expected weekly wet ion deposition associated with a one‐unit change in the predictor (e.g., a one‐day increase in smoke‐rain that week). To support interpretation, we generated marginal predictions from these models, which represent the expected value of the dependent variable (weekly wet deposition) from our gamma log regression model. Independent variables except the main predictor (smoke‐rain days) are held constant. Predictions are presented under reference conditions corresponding to summer 2022, with weekly precipitation held at its mean (27.8 mm).

Using the same modeling approach, we constructed regional models to understand variations in the relationship between smoke‐rain days and wet deposition across different regions. Models were built separately for climate regions with 5 or more unique NADP sites in a sample year, including Alaska & the Arctic and nine climate regions in the contiguous U.S. (Figure 1, Table S3). A Bonferroni correction was applied across regional models within each ion to adjust for multiple spatially stratified tests of the same hypothesis.

To understand the cumulative, annual impact of smoke‐rain days, we calculated the absolute and relative amount of smoke‐affected wet deposition to total observed deposition for each ion and study year. The gamma log‐link mixed effects “all sites” models were used to estimate rates of wet deposition for each week. A counterfactual prediction was generated for each site‐week: the expected wet deposition assuming zero smoke‐rain days (i.e., the baseline deposition level for that site under otherwise identical conditions). Wet deposition attributable to smoke‐rain was then calculated by taking the difference between the model‐estimated deposition under smoke‐rain and the counterfactual deposition under non‐smoke‐rain. Weekly wet deposition values for each ion and each year were summed across all sites to compute total observed deposition and total estimated smoke‐affected wet deposition. From these values, absolute and percent contribution of smoke‐affected wet deposition to total observed annual deposition were calculated.

To evaluate the potential effects of smoke‐affected wet deposition on ecosystems, we calculated the N:P (NH_4_^+^–N + NO_3_^−^–N/PO_4_^3−^–P) molar ratios of atmospheric wet deposition for each site‐week in the dataset. Following Isles (2020), N:P ratios were transformed using a natural logarithm. For each site, the log‐transformed ratios were averaged for (1) weeks without smoke‐rain (i.e., 0 smoke‐rain days), and (2) weeks with smoke‐rain (i.e., > 0 smoke‐rain days). Exponentiated ratios are presented for interpretability. All calculations and statistical analyses were performed in JMPv19 and Stata SE 18.0. Significance was set at *p* < 0.05.

## 3 Results

### 3.1 Spatial and Temporal Patterns of Smoke‐Rain Days

The frequency of smoke‐rain days at the NADP sites differed among the three focal years (2014, 2020, 2022) (Figure S3, Table S4). Across all NADP sites, 2014 had the fewest smoke‐rain days, on average (mean = 4, range = 0–20). The mean number of smoke‐rain days was slightly higher in 2020 (mean = 6, range = 0–19) than in 2014. Smoke‐rain days occurred most frequently in 2022 (mean = 21, range = 0–47). Although the maximum number of smoke‐rain days was similar for 2014 and 2020 (~20 days), a larger proportion of NADP sites was affected by smoke‐rain in 2020 compared to 2014. Smoke‐rain days were recorded at 82% of NADP sites in 2014 compared to 96% of NADP sites in 2020. In 2022, 25% of all NADP sites recorded between 30 and 47 smoke‐rain days.

**FIGURE 2:**
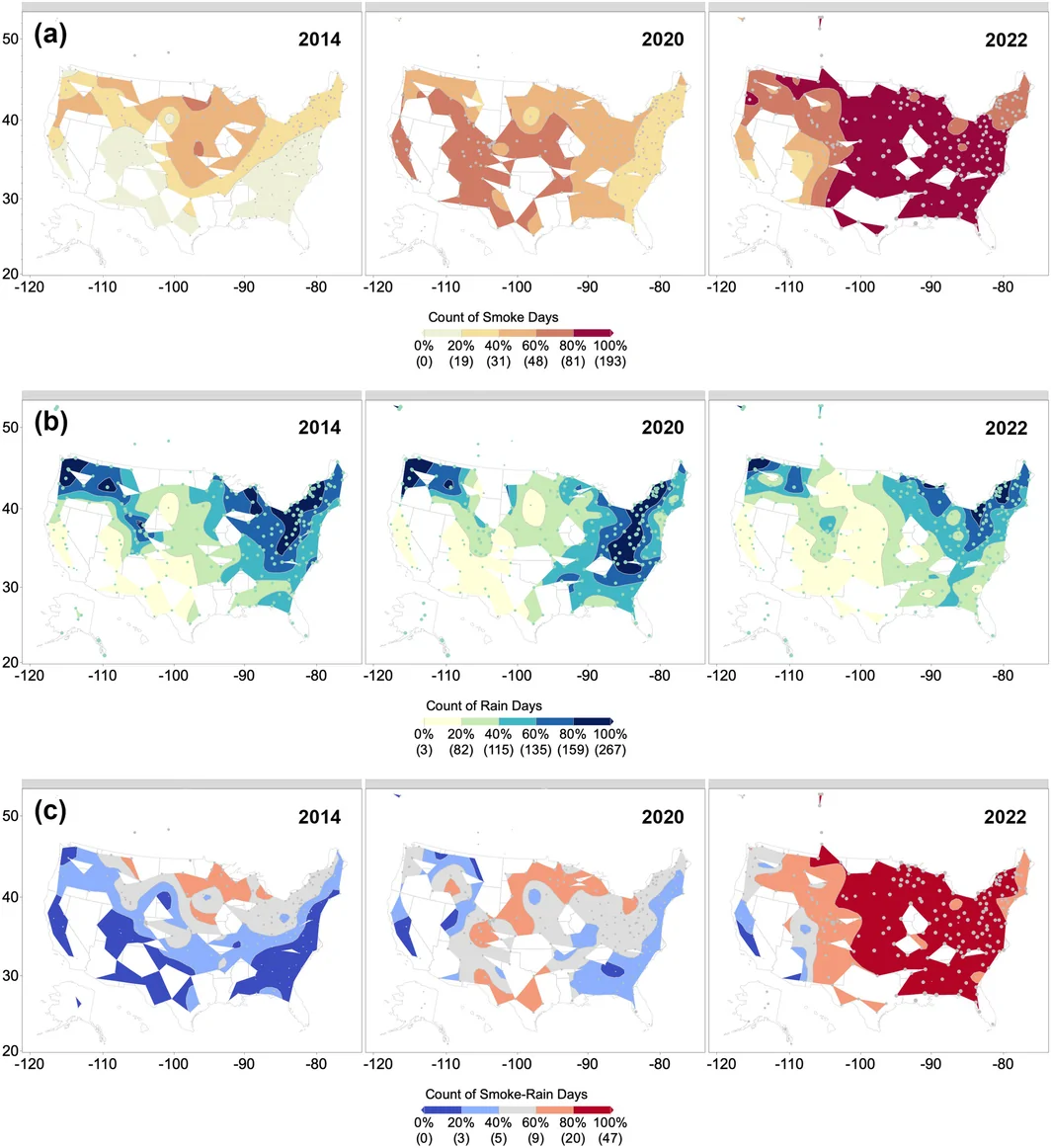
Contour plots interpolated by Delauney triangulation showing count of (a) smoke days, (b) rain days, (c) smoke‐rain days at the U.S. NADP sites for the years 2014, 2020, and 2022. Percentages are quantiles. Dots represent NADP sites and are sized proportional to counts. Map lines delineate study areas and do not necessarily depict accepted national boundaries.

The spatial distribution of smoke‐rain days also differed markedly among the study years (Figure 2c, Table S4). In 2014, smoke‐rain days were most frequent at NADP sites in the Upper Midwest, Northern Rockies, and Northeast regions. In 2020, there was a similar number of smoke‐rain days at sites across most regions in the contiguous U.S. (mean = 5–9 per region), with the highest number of days in the Upper Midwest and Southwest regions. In contrast, there were few smoke‐rain days in western U.S. and Canadian climate regions (West, Alaska & the Arctic, South British Columbia Mountains, Pacific Coast, Prairies) because despite high smoke frequency, smoke did not occur when there was also rain. Smoke‐rain days were frequent (mean = 18–33 per region) across most regions (9 of 16) in 2022, with the most smoke‐rain days in the Upper Midwest, Ohio Valley, and Southeast. The contour plots show that the number of smoke days (Figure 2a) affecting NADP sites was highly variable across years while the number of rainfall days (Figure 2b) was not.

In every year, smoke‐rain days were concentrated in the months of July, August, and September. However, in 2022, smoke‐rain days occurred from spring to fall—March to October—consistent with reports of longer fire seasons (Cattau et al. 2020). In sum, at the majority of NADP sites, there was at least one smoke‐rain day in any given year; the occurrence of smoke‐rain days varied spatially among study years and was more driven by variation in annual smoke (Figure 2a) than annual rainfall (Figure 2b) at the NADP sites; and smoke‐rain days occurred most frequently in July, August, and September during the active growing season.

### 3.2 Smoke‐Rain Effects on Wet Ion Deposition

**FIGURE 3:**
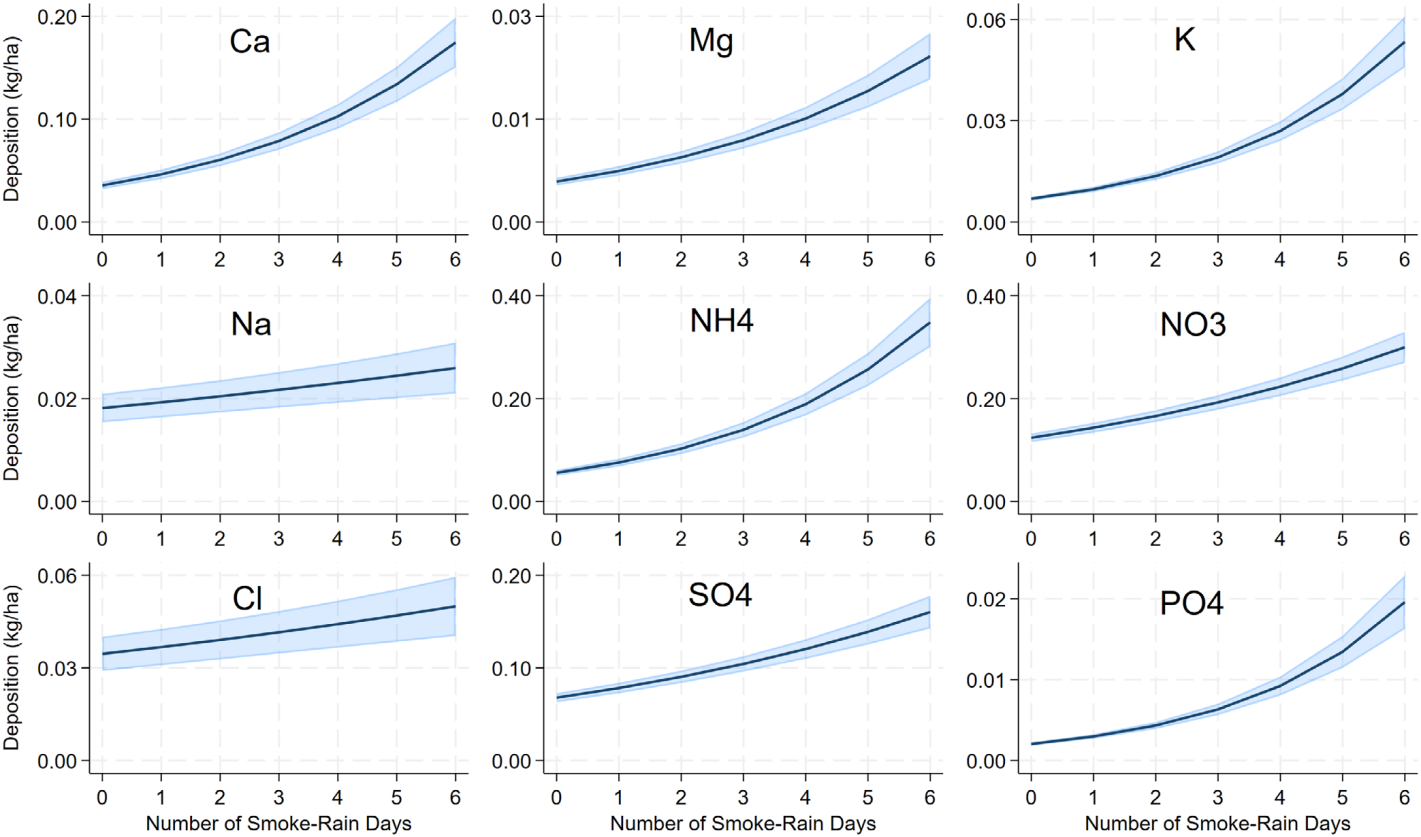
Marginal predictions of expected weekly wet deposition (kg/ha) for nine ions as a function of the number of smoke‐rain days per week calculated from mixed gamma regression models with a log link. Shaded bands indicate 95% confidence intervals.

Based on estimates derived from the marginal predictions of the “all sites” model, across all NADP sites and study years, smoke‐rain days had significant positive effects on the weekly wet deposition of the nine ions evaluated (Figure 3, Table S5). Depending on the ion, wet deposition increased anywhere from 6% to 46% with each additional smoke‐rain day per week. The largest increases in weekly deposition (for 1–6 smoke‐rain days) were observed for PO_4_^3−^ (46%, 0.003–0.02 kg/ha/week), K^+^ (41%, 0.01–0.053 kg/ha/week), and NH_4_^+^ (36%, 0.076–0.348 kg/ha/week). Effects of smoke‐rain on wet deposition were weakest for Na^+^ (6%) and Cl^−^ (6%).

**FIGURE 4:**
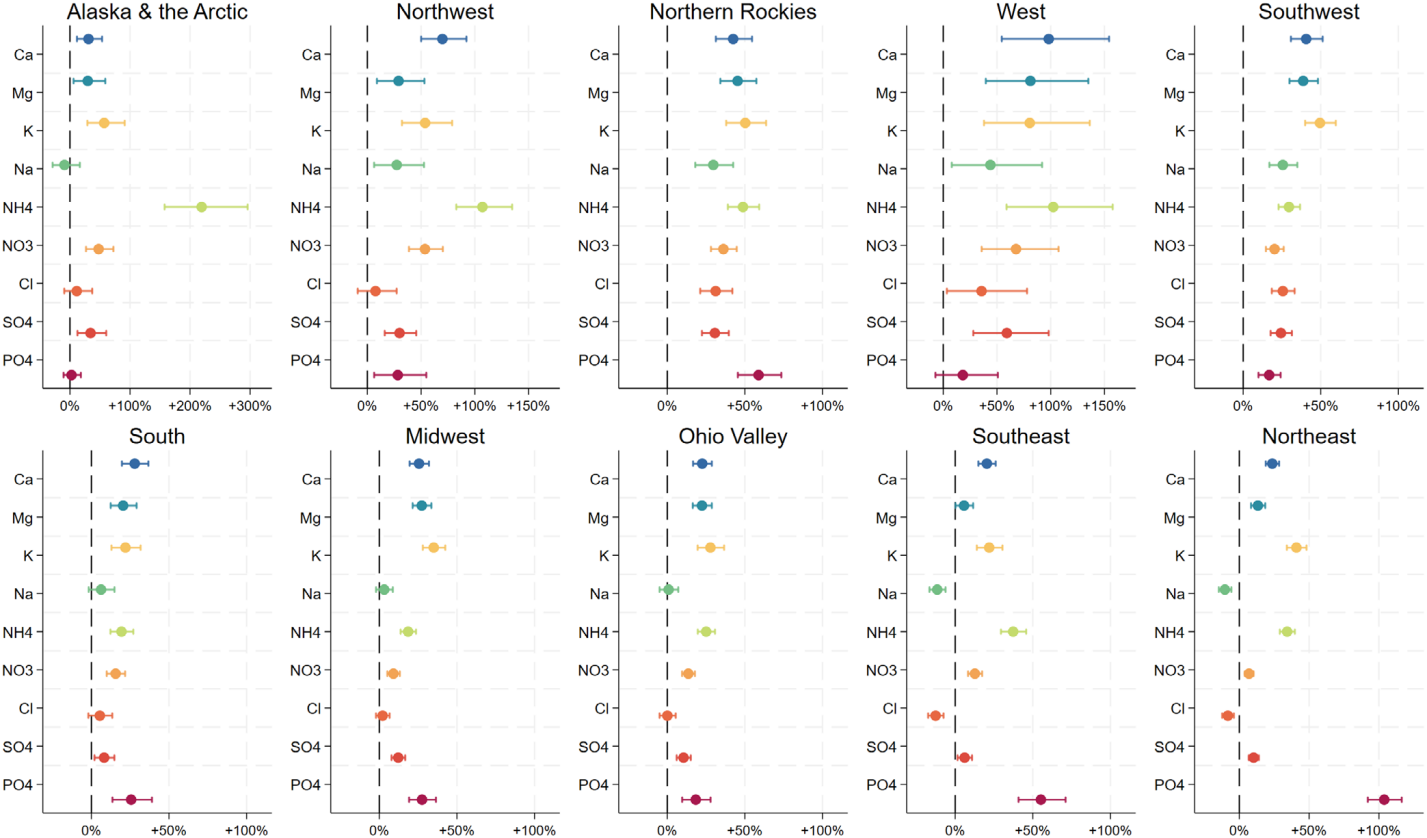
Regional estimates of the percent change in modeled weekly wet deposition associated with a one‐day increase in the number of smoke‐rain days within a week based on mixed gamma regression models with a log link. Models were fit separately for each climate region and adjusted for seasonal and interannual variation in deposition, as well as precipitation level. Points show exponentiated coefficients expressed as percent change, and horizontal bars represent 95% confidence intervals.

Smoke‐rain effects on wet deposition varied by region, both in terms of magnitude and composition (Figure 4; Table S6). Alaska & the Arctic, the Northwest, and West climate regions experienced significant increases in the weekly wet deposition of several ions as a result of smoke‐rain, particularly NH_4_^+^ (102%–218%) and K^+^ (54%–81%), and Ca^2+^ in the Northwest and West (70%–98%). Greater uncertainty is associated with these estimates relative to other regions, however, especially in the West (Figure 4). In the Northern Rockies and Southwest regions, smoke‐rain led to significant increases in the deposition of all nine ions, and the confidence intervals were narrow. In the Northern Rockies, deposition was elevated most for PO_4_^3−^ (57%), K^+^ (49%) and NH_4_^+^ (48%) compared to non‐smoke‐rain days. Smoke‐rain days contributed to elevated wet deposition of K^+^, Ca^2+^, and Mg^2+^ in the Southwest (49%, 41%, 39%, respectively) and Upper Midwest (35%, 26%, 27%, respectively). Smoke‐rain effects on wet PO_4_^3−^ deposition were pronounced in the Northeast and Southeast, where each additional smoke‐rain day was associated with ~104% and 55% more PO_4_^3−^, respectively. Compared to other climate regions, the Ohio Valley and South showed weaker effects (i.e., < 30% increase) of smoke‐rain on atmospheric wet deposition as did the Upper Midwest, despite the latter region experiencing frequent smoke‐rain days across study years.

**FIGURE 5:**
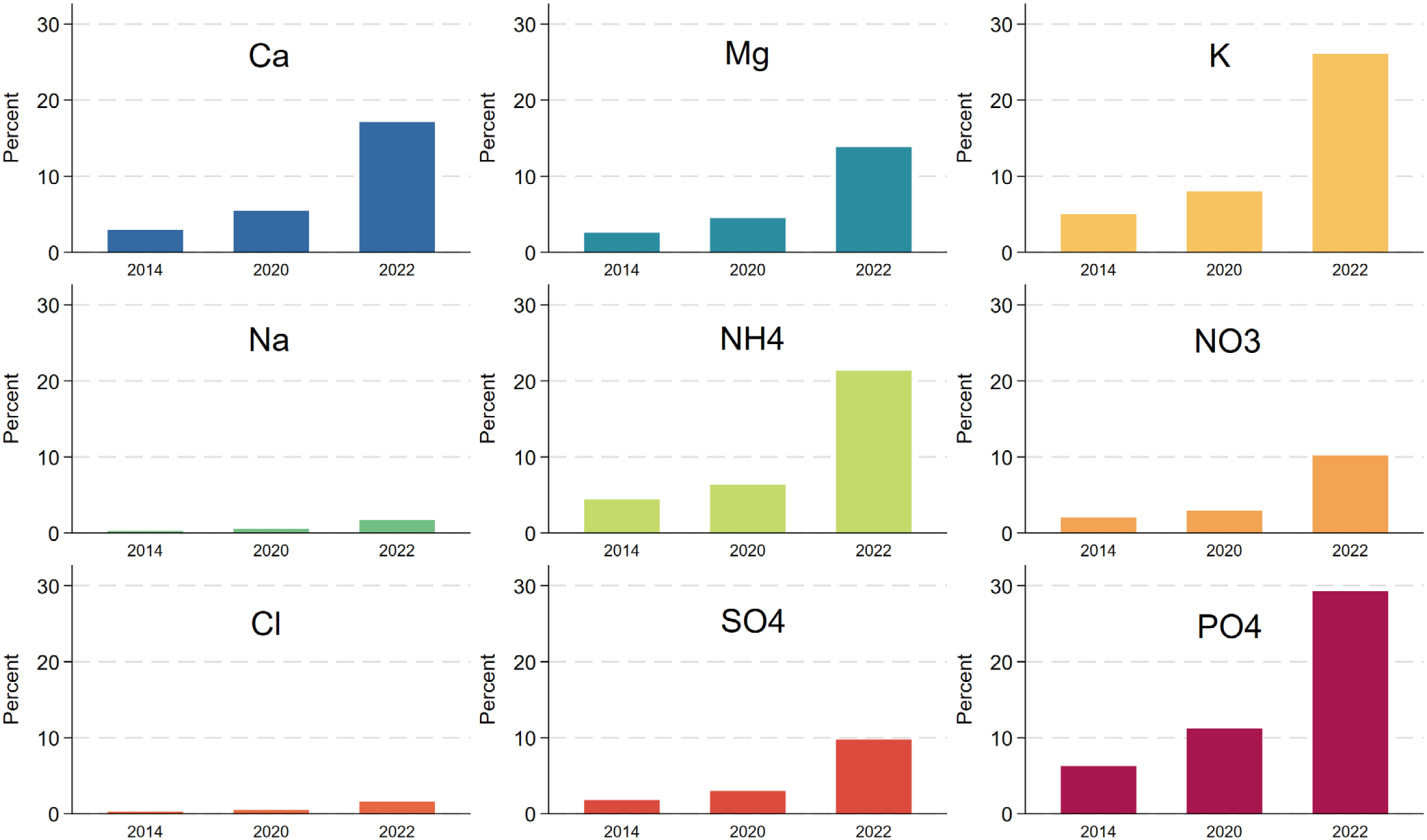
The percentage of total annual wet deposition attributable to smoke‐rain for each ion across the three study years based on estimates from mixed gamma regression models with a log link.

Results show that depending on the ion and year, smoke‐rain accounts for anywhere from < 1% (for Na^+^) to nearly 30% of estimated annual wet deposition (for PO_4_^3−^). All else equal, the percent of annual wet deposition attributable to smoke‐rain is greater in years with higher smoke‐rain frequency (Figure 5). In 2022, when smoke‐rain affected NADP sites, on average, 21 days per year, smoke‐rain accounted for nearly one‐third of annual wet deposition of PO_4_^3−^ (0.036 kg/ha/year) and K^+^ (0.082 kg/ha/year) compared to ~5% (0.006 kg PO_4_^3−^/ha/year, 0.014 kg K^+^/ha/year) and ~10% (0.015 kg PO_4_^3−^/ha/year, 0.024 kg K^+^/ha/year) of annual wet deposition in 2014 and 2020, respectively. For NH_4_^+^, we estimate that the share of annual wet deposition attributable to smoke‐rain days was as much as 20% in 2022 (0.458 kg NH_4_^+^/ha/year). By comparison, wet deposition from smoke‐rain was much lower in 2014 (0.095 kg NH_4_^+^/ha/year) and 2020 (0.132 kg NH_4_^+^/ha/year). The influence of smoke rain on the other evaluated ions is reported in Text S1.

### 3.3 Smoke‐Rain Effects on Molar N:P Ratios

**FIGURE 6:**
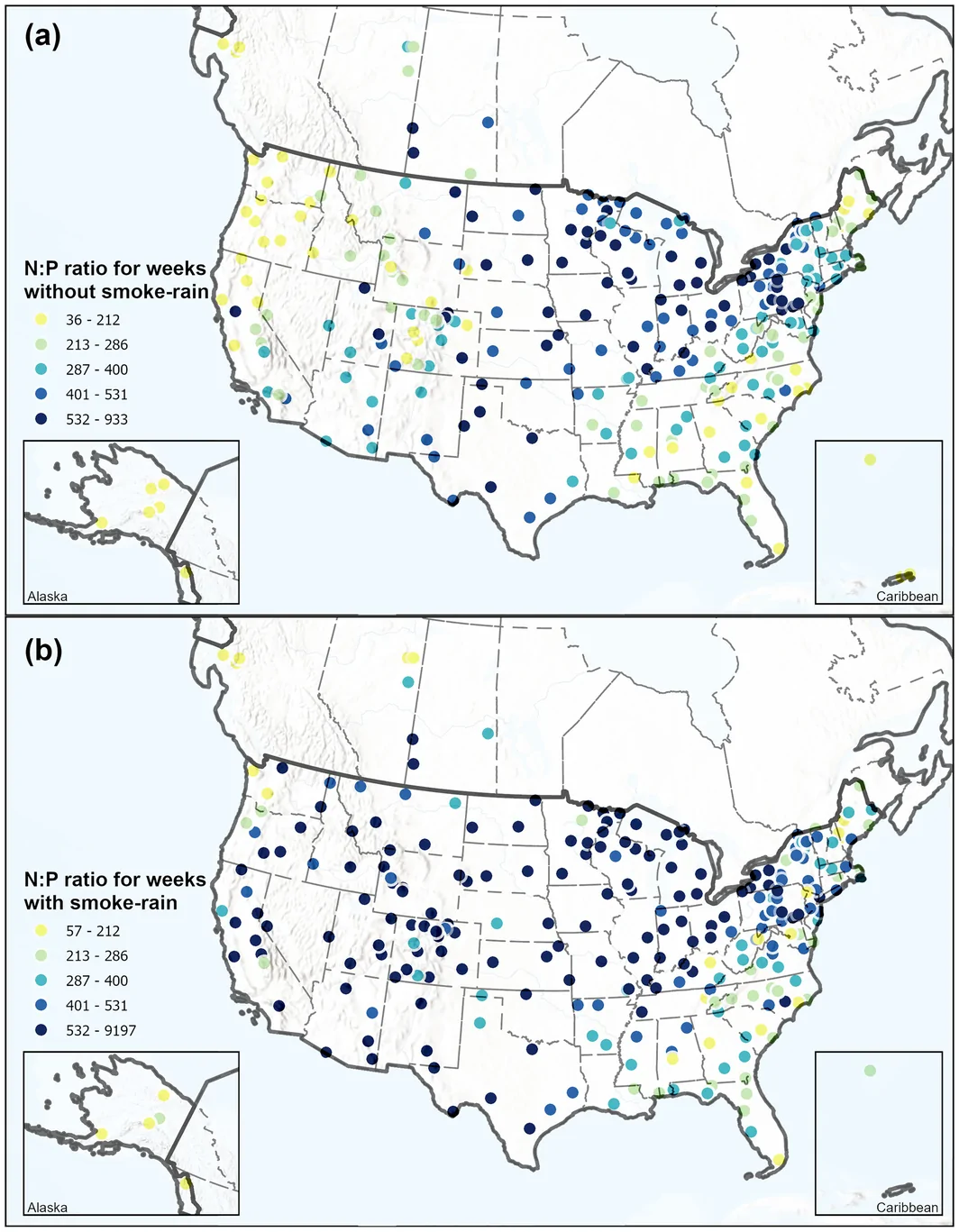
Weekly molar N:P ratios (exponentiated) of atmospheric wet deposition at NADP sites during (a) weeks without smoke‐rain days, and (b) weeks with smoke‐rain days. Map lines delineate study areas and do not necessarily depict accepted national boundaries.

We calculated molar N:P ratios of observed wet deposition to assess the potential ecosystem effects of smoke‐affected wet deposition. During weeks without any smoke‐rain days, logged N:P ratios ranged from 3.57 to 6.84 (mean = 5.76, exponentiated = 316). Ratios where higher during weeks affected by smoke‐rain: the range was 4.01–9.13 (mean = 6.2, exponentiated = 493). There was substantial variability at the site level, however. Molar N:P ratios were higher at 76% (*n* = 217 sites) and lower at 19% (*n* = 53 sites) of NADP sites during weeks with (Figure 6b)—compared to weeks without (Figure 6a)—smoke‐rain days. Molar N:P ratios increased more in western U.S. climate regions, including the West, Northwest, Southwest, Northern Rockies, and Alaska & the Arctic compared to other regions. Forty percent of the sites where molar ratios decreased were in the Northeast, whereas 28% were in the Southeast and Ohio Valley climate regions. The magnitude of the decrease in N:P ratios was similar among regions.

## 4 Discussion

### 4.1 Wildland Fires Could Fertilize Terrestrial and Aquatic Ecosystems

Wildland fires are a major and growing emitter of gases and particles to the atmosphere in extratropical regions (Jones et al. 2024). A recent multi‐year, national‐scale study found that wildland smoke PM_2.5_ emissions enhance ambient concentrations of a wide range of chemical species on smoke days, with K and P showing the largest concentration increases relative to non‐smoke days (Krasovich Southworth et al. 2025). Our analysis confirms that when wildland smoke is mixed with and deposited in rain, it is also a non‐negligible source of biologically important nutrients to ecosystems.

Smoke drives increased wet deposition of essential macronutrients that limit growth and production in terrestrial and aquatic ecosystems. Depending on the year, smoke‐affected rain can contribute up to 0.45 kg/ha/year of inorganic N (NH_4_^+^–N + NO_3_^−^–N), 0.04 kg/ha/year of PO_4_^3−^, 0.08 kg/ha/year of K^+^, 0.24 kg/ha/year of Ca^2+^, 0.04 kg/ha/year of Mg^2+^, and 0.28 kg/ha/year of SO_4_^2−^. Further, in a high smoke year, such as 2022, smoke‐rain days can deliver a major proportion (~20%–30%) of annual wet dissolved deposition for several of these key elements. Notably, our estimates are lower in magnitude than measured dry dust‐derived nutrient fluxes (Brahney, unpublished; Lawrence et al. 2010). For example, across sites in Utah and Wyoming, dust‐deposited Ca is ~1.04 kg/ha/year (CI: 0.75–1.34 kg/ha/year; Brahney, unpublished) whereas in this study Ca attributable to smoke‐rain at U.S. sites is 0.24 kg/ha/year (CI: 0.10–0.13 kg/ha/year). Yet despite the differences in magnitude, nutrients in smoke‐affected wet deposition are more pervasive, impacting areas that both do and do not normally experience dust deposition.

The absolute and relative magnitudes of wildland smoke deposition are in fact likely to be much larger because the estimates we present here do not include dry deposition on smoke days, which occur far more frequently than smoke‐rain days. At the NADP sites, there were 5 times (in 2014), 7 times (in 2020), and 4 times (in 2022) more smoke‐ than smoke‐rain days (Table S7). Dry inputs can comprise a significant fraction of the total material deposited to ecosystems, and modeling studies show that, at least for N, fire emissions contribute more to dry than to wet deposition (e.g., Koplitz et al. 2021; Campbell et al. 2022, 2026). Campbell et al. (2026) further show that dry N deposition represents an increasing proportion of total N deposition in the Northwest and West regions. Nonetheless, our wet deposition estimates are substantial given that on average smoke‐rain days made up 1%–5% of the year.

Although we focused on the effects of smoke‐rain days on the wet deposition of dissolved nutrients, wildland fires also emit substantial amounts of ash (combustion particles in the larger size fractions) to the atmosphere. The positive relationships we found between charcoal on sample filters and the wet deposition of Ca^2+^, Mg^2+^, K^+^, NH_4_^+^, NO_3_^−^, and PO_4_^3−^ underscore the importance of measuring and accounting for larger particles in wet nutrient deposition (Ponette‐González et al. 2018; Brahney et al. 2024). The same is of course true for dry deposition of ash‐associated nutrients that may become available after deposition. For instance, larger fire‐emitted particles that deposit directly to water bodies contain nutrients that can become immediately bioavailable upon contact with water or after oxidation (Chandra et al. 2022). Ash aerosols collected in the Santa Barbara Basin during the Thomas Fire in California, U.S., were analyzed for trace metals and had water leachable fractions ranging from 61% for zinc to 6% for iron (Kelly et al. 2021). Chandra et al. (2022) also measured water extractable nutrients in ash, but from the 2021 Caldor Fire in California, U.S. They found that nutrient concentrations differed with the degree of ash pyrolization, which in turn had effects on production and respiration in microbial and algal communities through nutrient enrichment, demonstrating the effect of ash quality on ecosystem outcomes. In sum, additional measurements of dry deposition, improved representation of dry deposition processes within chemical transport models (Koplitz et al. 2021), and measurements of particulates in deposition across the relevant size spectra (> 10 μm) are needed to more accurately quantify wildland smoke emission impacts on downwind deposition (Brahney et al. 2015; Farruggia et al. 2024; Scordo et al. 2026).

### 4.2 Regional Patterns of Smoke‐Affected Wet Deposition

Overall, smoke‐affected rain showed the largest wet deposition enhancement for N, P, K, and Ca, yet there were clear regional differences in the magnitude of these effects. We observed patterns in the chemistry of wet deposition that are consistent with reported regional impacts of smoke on ambient concentrations and atmospheric deposition.

In the U.S., regional wildfire patterns have changed since 2000 (Iglesias et al. 2022). The Eastern region(s) experiences more fires, whereas the Great Plains and West experience more fires, more area burned, and more extreme fires than in earlier decades (1984–1998). Our analysis reveals that during the study years, smoke‐rain effects on wet deposition were larger in western U.S. climate regions (Alaska & the Arctic, Northwest, Northern Rockies, West, and Southwest) than in eastern climate regions (Northeast, Southeast, Ohio Valley Upper Midwest, South) and for nearly all ions. However, there was also more uncertainty around these regional estimates, which could be due to higher site‐to‐site variation in smoke‐affected wet deposition and/or the fewer number of NADP sites affected by smoke‐rain within these western climate regions. Regardless, given the current trend of increasing western U.S. wildfire activity (Westerling 2016; reviewed in Jones et al. 2022), there is a need to better quantify and characterize spatial variability in smoke‐affected deposition across this region.

In Alaska & the Arctic, the Northwest, and West, weekly increases in NH_4_^+^ with smoke‐rain ranged from 102% to 218% per additional day of smoke‐rain. Wildfires are a major source of NH_3_ to the atmosphere in the western U.S. (Benedict et al. 2017; Lindaas et al. 2021) and, in a very recent study, Campbell et al. (2026) reported that increasing wildfire contributions to N deposition in the Northwest U.S. were largely due to increases in dry deposition of reduced N (NH_4_^+^ and NH_3_). In Alaska, Nagano and Iwata (2017) found that burned area near and far from a dry deposition monitoring site was an important predictor of measured dry N deposition. Although that study did not distinguish the contribution of smoke from fires to oxidized and reduced N, most fires in Alaska occur in boreal forest and are characterized by high NH_3_ emissions (Chen et al. 2025). These studies suggest that smoke from fires, and forest fires in particular, may also be contributing to elevated wet deposition of reduced N in western U.S. regions.

Increases in wet deposition of K^+^ with smoke‐affected rain were most pronounced in the West as well (81%, Table S6). Potassium is often considered a good inorganic tracer of biomass burning in aerosol because of its abundance in plant tissues. However, K emissions vary with fuel type and combustion conditions (flaming vs. smoldering) (Hays et al. 2002), and seasalt and mineral dust represent additional sources of K^+^. Thus, the strength of this tracer can be expected to vary regionally (e.g., see Bian et al. 2020). In the western U.S., active sampling of fire plumes has shown the prevalence of K in fire‐emitted particles (Silva et al. 1999; Hudson et al. 2004). In addition, Bian et al. (2020) found that non‐seasalt K concentrations in fine particles were higher in the Pacific West compared to the Southeast, a pattern they attributed to different burn conditions in these regions (i.e., more flaming fires in the West and more smoldering fires in the Southeast).

The Northwest and West also displayed substantial enhancements in Ca^2+^ deposition with wildland smoke compared to other regions. Forest fires could explain the elevated levels of Ca^2+^ we detected in smoke‐affected wet deposition (Brahney et al. 2013), or alternatively, the increased Ca^2+^ may be evidence of dust entrained in fire plumes (Wagner and Schepanski 2025). Schlosser et al. (2017) analyzed particulate matter concentrations from U.S. IMPROVE sites and found that fine soil and coarse PM were enhanced during peak fire periods indicative of dust. During these peak fire periods, California and the northwestern states of Oregon and Washington showed the largest relative increases in Ca^2+^ in particulate matter, which aligns with our findings.

In eastern U.S. climate regions, PO_4_^3−^ displayed the most substantial increase in wet deposition with smoke‐rain days. In the Northeast, each additional smoke‐rain day was associated with ~104% more PO_4_^3−^. One potential source of P in wet deposition in this region is Canadian wildfire smoke. Fernandez et al. (2024) sampled rainwater at a northeastern site downwind of active Canadian wildfires and found that dissolved phosphorus exhibited the strongest signal of smoke in rainwater samples. The relative contribution of smoke‐rain days to PO_4_^3−^ was less strong in the Southeast (55%) than in the Northeast but notable compared to other regions. The Southeast accounts for ~60% of all prescribed fires in the U.S. (Melvin 2020). In addition to forestry/rangeland burning in this region, agricultural burning is a major source of atmospheric P (Wang et al. 2015) that could be influencing wet deposition in this region.

Although the Upper Midwest emerged as a persistent hotspot for smoke‐rain, there were only modest increases in smoke‐affected wet deposition in this region. This raises the question: what drives regional variation in the magnitude and composition of smoke‐affected wet deposition? As this example highlights, several factors in addition to smoke‐rain day frequency influence wet deposition during smoke periods, including fire characteristics and behavior, distance from fire, and the type of material burned (Chandra et al. 2022; Scordo et al. 2026). During combustion, different vegetation types (e.g., trees, grasses, shrubs) emit particulates that vary in their chemical composition (Urbanski 2014), affecting the chemical signature of smoke‐affected rainwater and deposition downwind. A region's distance to fire can alter wet deposition chemistry as well. Distance affects the time available for oxidation, photochemistry, and other reactions to occur within smoke plumes during transport, leading to differences in composition between aged and fresh smoke. Fire properties which vary regionally, such as combustion temperature, determine the extent to which different nutrients are volatilized and thus their relative abundance in smoke and ash (Bodí et al. 2014; Chandra et al. 2022). Fire heat can even affect precipitation patterns at regional scales driving increased wet deposition downwind of fire (Ma et al. 2025). Thus, a region's dominant vegetation type, location relative to smoke sources, and typical fire regime likely play important roles in explaining regional patterns of smoke‐affected wet deposition. We speculate that regional patterns will shift over time in tandem with the changing geography of wildland smoke, vegetation and reburn, and altered rainfall patterns. Our data suggest, for example, that the relative importance of smoke‐rain is likely to be higher in regions with summer rainfall as opposed to summer drought.

### 4.3 Implications for Downwind Ecosystems

In relative terms, smoke‐rain days had the largest estimated impact on wet PO_4_^3−^ deposition, underscoring the importance of wildland smoke as a source of bioavailable P. These findings support a growing body of evidence showing the potential for high fire‐derived P deposition to forest (Boy et al. 2008; Ponette‐González et al. 2016; Bauters et al. 2021), aquatic (Brahney et al. 2015; Fernandez et al. 2024; Olson et al. 2026), and marine (Barkley et al. 2019; Tang et al. 2021) systems both near and far downwind of burning vegetation.

Across the NADP network, annual wet PO_4_^3−^–P deposition ranged from 3.1 to 4.3 mg/m^2^/year (mean = 3.2 mg/m^2^/year). These values are substantially lower than the mean PO_4_^3−^–P deposition rate (19 mg/m^2^/year) reported by Tipping et al. (2014) for 11 North American sites where direct measurements of PO_4_^3−^–P were conducted between 1954 and 2012, but they are well within the range of potential ecosystem impacts (Scholz and Brahney 2022). Moreover, we analyzed wet‐only P deposition, whereas Tipping et al. (2014) synthesized bulk deposition estimates, which are typically larger than wet‐only estimates due to particulate deposition to bulk samplers during dry periods.

Smoke‐affected wet PO_4_^3−^–P deposition ranged from 0.2 to 1.2 mg/m^2^/year across the study years and averaged 0.62 mg/m^2^/year. Thus, the fraction of PO_4_^3−^–P attributable to smoke‐rain was ~16% of annual wet PO_4_^3−^–P deposition. In a global modeling study, Mahowald et al. (2008) estimated that biomass burning constitutes ~5% of wet plus dry PO_4_ deposition. The NADP observational dataset suggests that smoke has a larger impact on rain chemistry in the U.S. and Canada or that the impact is increasing over time. Investigation of smoke‐affected wet P deposition over a longer time period is needed to address this question.

Dust is the largest global source of atmospheric P (Mahowald et al. 2008). Thus, it is not surprising that PO_4_^3−^–P deposition from smoke is often lower than reported contributions from atmospheric dust, especially in semi‐arid to arid ecosystems where overall dust deposition rates are high (Brahney et al. 2024). For example, our estimate of smoke‐affected wet PO_4_^3−^–P deposition is lower than the average dust deposition rate of bioavailable P (1.6 mg P/m^2^/year) reported by Brahney (2019) and the wet plus dry dust deposition of bioavailable P reported by Scholz and Brahney (8.8 mg/m^2^/year), both for northern Utah. Our estimate is also lower than dust P deposition measured in the Sierra Nevada Mountains of California (3.6–19 mg/m^2^/year; Aciego et al. 2017). However, the mean value of 0.62 mg P/m^2^/year in smoke‐affected wet deposition is similar in magnitude to short‐term soil production rates from P‐rich bedrock reported in that study (i.e., 0.2–4.9 mg/m^2^/year, mean = 1.7 mg/m^2^/year). We present annual averages for comparison but note that the maximum rate of smoke‐affected wet PO_4_^3−^–P deposition estimated at an NADP site was 12.5 mg/m^2^/year (2014), 28.1 mg/m^2^/year (2020), and 26.4 mg/m^2^/year of PO_4_^3−^–P (2022). Thus, there are sites in the NADP network where PO_4_^3−^–P delivered in smoke‐affected rain is much higher than bioavailable P in dust and 7‐fold to 17‐fold higher than P release from soil weathering (Aciego et al. 2017).

Even so, the overall estimates we present here for NADP sites are likely conservative. The NADP network measures orthophosphate in rainwater to determine potential sources of contamination and these inorganic P measurements could underestimate the total flux of bioavailable P in precipitation for several reasons. First, no acid or biocide is used in rainwater samples to prevent uptake into organisms or limit exchanges with the plastic sample containers. Second, low volume samples are diluted before analysis, resulting in higher detection limits. Third, the samples are not analyzed using techniques that are standard for soluble reactive phosphorus or total phosphorus measurements, which are commonly used to understand freshwater impacts. Additionally, not all forms of contamination are reported or observed, and because of the generally low values (90% of valid samples are below the MDL), the precision is expected to be no greater than ±100%. Despite these caveats, the strong influence of smoke‐rain on P deposition we report highlights the need for routine monitoring of P in atmospheric deposition monitoring networks in the U.S. and other regions.

The shifts in molar N:P ratios also indicate the potential for changes in the severity of ecosystem nutrient limitation. During weeks with and without smoke‐rain, site‐level molar N:P ratios of atmospheric wet deposition were always above the Redfield ratio of 16:1 and N:P ratios for forest foliage (28) and senesced litter (45) (McGroddy et al. 2004). However, for most sites (76%), weeks affected by smoke‐rain led to significantly higher molar N:P ratios, especially in the western half of the U.S. Smoke‐rain deposition could thus exacerbate P limitation in P‐limited systems, such as western U.S. montane forests and most lakes. Very recent studies nevertheless suggest that atmospheric P inputs to lakes during smoke events (Olson et al. 2023; Fernandez et al. 2024) can increase surface chlorophyll‐a concentrations (Olson et al. 2026). Olson et al. (2026) found that these effects were most pronounced in high elevation lakes and in the Upper Midwest, where our data show higher frequency of smoke and smoke‐rain days.

The frequency and magnitude of extreme wildfire events are increasing globally (Cunningham et al. 2024), including in many U.S. regions (Lindley et al. 2019; Iglesias et al. 2022). It is reasonable to speculate that if smoke‐rain deposition increases in tandem with increases in smoke PM, fire‐enhanced nutrient deposition could fertilize downwind ecosystems with potential for cascading ecological effects in both terrestrial and aquatic systems. As such, future wildfires and their emissions may fertilize land and water.

## Author Contributions

**Alexandra G. Ponette‐González:** conceptualization, methodology, data curation, supervision, funding acquisition, visualization, writing – original draft, writing – review and editing. **Janice Brahney:** conceptualization, methodology, data curation, supervision, funding acquisition, writing – original draft, writing – review and editing. **Bryn Spielvogel:** data curation, formal analysis, methodology, visualization, writing – original draft. **Heather A. Holmes:** methodology, conceptualization, writing – review and editing. **Kathleen C. Weathers:** conceptualization, methodology, writing – review and editing.

## Funding

Funding for this research was provided the U.S. National Science Foundation (DEB 2320976 to Ponette‐González, CBET 2048423 to Holmes) and by the University of Utah Vice President for Research Faculty Small Grant Program. The research was also supported by the Utah Agriculture Experiment Station (UTA01669, Brahney), approved as journal paper number UAES 9996, and the U.S. National Science Foundation (DEB 2213625 to Brahney).

## Conflicts of Interest

The authors declare no conflicts of interest.

## Supporting information


**Figure S1:** Cumulative smoke distribution for the contiguous U.S. derived from the Hazard Mapping System (HMS) fire and smoke product. Data show the total number of days when smoke covered the U.S. Contour lines depict spatial variability in the number of observed smoke days. Data accessed February 1, 2026, at https://www.ospo.noaa.gov/Products/land/hms.html.
**Figure S2:** Significant relationships between nutrient deposition (kg/ha/week) and charcoal deposition counted on sample filters for 36 filters collected at 5 sites in the western U.S. in summer 2020. Potassium, PO_4_^3−^, and Mg^2+^ deposition exhibited moderate relationships (K^+^: *R* ^2^ = 0.56, *p* < 0.0001; PO_4_^3−^: *R* ^2^ = 0.38, *p* = 0.0005; Mg^2+^: *R* ^2^ = 0.34, *p* = 0.0012), whereas Ca^2+^, NH_4_^+^, and NO_3_^−^ exhibited weak relationships (Ca^2+^: *R* ^2^ = 0.20, *p* = 0.0174; NH_4_^+^: *R* ^2^ = 0.29, *p* = 0.0031; NO_3_^−^: *R* ^2^ = 0.15, *p* = 0.0454) with charcoal on the sample filters.
**Figure S3:** Distribution of smoke‐rain days (i.e., when smoke and rainfall occur at the site on the same day) at National Atmospheric Deposition Program (NADP) sites in 2014, 2020, and 2022.
**Table S1:** National Atmospheric Deposition Program (NADP) wet deposition samples (*n* = 891) included in the dataset and labeled with invalcode “c.” Breakdown includes number and percentage of samples in the dataset and broken out by climate region.
**Table S2:** Criteria used by the National Atmospheric Deposition Program (NADP) and in this study to exclude cases from the analytic file.
**Table S3:** Climate regions with five or more unique National Atmospheric Deposition Program (NADP) sites in a sample year used to build regional mixed effects models of smoke‐rain effects on wet deposition.
**Table S4:** Descriptive statistics of total annual smoke‐rain days by season, climate region, and by year.
**Table S5:** Marginal predictions of expected weekly wet deposition for nine ions across 0–6 smoke‐rain days per week. Predictions are derived from gamma regression models with a log link and are evaluated under reference conditions corresponding to summer 2022, with weekly precipitation held at its mean (27.8 mm).
**Table S6:** Multiplicative effects of smoke‐rain days on wet deposition, expressed as exponentiated coefficients (exp (*β*)), from gamma mixed‐effects models. Coefficients represent multiplicative differences in expected weekly ion wet deposition as the number of smoke‐rain days within a week increases, holding weekly precipitation amount, season, and year constant and accounting for repeated measurements within sites. Values greater than 1 indicate higher expected deposition in weeks with more smoke‐rain days relative to non‐smoke‐rain days (e.g., exp (*β*) = 1.30 corresponds to 30% higher expected deposition), whereas values less than 1 indicate lower expected deposition (e.g., exp (*β*) = 0.88 corresponds to 22% lower expected deposition).
**Table S7:** Descriptive statistics of total annual smoke days by season, climate region, and by year.
**Text S1:** Wet deposition attributable to smoke‐rain for Ca^2+^, Mg^2+^, SO_4_^2−^, NO_3_^−^, Na^+^, Cl^−^ at National Atmospheric Deposition Program (NADP) sites for the years 2014, 2020, and 2022.

## Data Availability

The data underlying this study are available on the HIVE repository at https://doi.org/10.7278/S5d‐0t43‐qwx6.
