# Supplementary material for: Smoke‐Affected Rain Fertilizes Terrestrial and Aquatic Ecosystems
Source: Glob Chang Biol. 2026 Aug 25;32(8):e71050. doi: 10.1111/gcb.71050 (PMC13504409; doi:10.1111/gcb.71050)
Supplement: Supplementary file 1 [file GCB-32-e71050-s001.pdf]

Supporting Information

Smoke-affected rain fertilizes terrestrial and aquatic ecosystems

A.G. Ponette-González[1,2,*], J. Brahney[3], H.A. Holmes[4], B. Spielvogel[5], K.C. Weathers[6]

[1]Natural History Museum of Utah, University of Utah, Salt Lake City, UT, USA
[2]Department of City & Metropolitan Planning, University of Utah, Salt Lake City, UT, USA
[3]Department of Watershed Sciences and Ecology Center, Utah State University, Logan, Utah, USA
[4]Department of Chemical Engineering, University of Utah, Salt Lake City, UT, USA
[5]Department of Parks, Recreation, and Tourism, University of Utah, Salt Lake City, UT, USA
[6]Cary Institute of Ecosystem Studies, PO Box AB, Millbrook, NY 12545

This file contains the following figure and tables.

Text S1. Wet deposition attributable to smoke-rain for Ca^{2+}, Mg^{2+}, SO_4^{2-}, NO_3^-, Na^+, Cl^- at National Atmospheric Deposition Program (NADP) sites for the years 2014, 2020, and 2022.

Fig. S1. Cumulative smoke distribution for the contiguous U.S. derived from the Hazard Mapping System (HMS) Fire and Smoke Product. Data show the total number of days when smoke covered the U.S. Contour lines depict spatial variability in the number of observed smoke days. Data accessed February 1, 2026, at https://www.ospo.noaa.gov/Products/land/hms.html

Fig. S2. Significant relationships between nutrient deposition (kg/ha/wk) and charcoal deposition counted on sample filters for 36 filters collected at 5 sites in the western U.S. in summer 2020.

Fig. S3. Distribution of smoke-rain days (i.e., when smoke and rainfall occur at the site on the same day) at National Atmospheric Deposition Program (NADP) sites in 2014, 2020, and 2022.

Table S1. National Atmospheric Deposition Program (NADP) wet deposition samples (n=891) included in the dataset and labeled with invalcode “c”. Breakdown includes number and percentage of samples in the dataset and broken out by climate region.

Table S2. Criteria used by the National Atmospheric Deposition Program (NADP) and in this study to exclude cases from the analytic file.

Table S3. Climate regions with 5 or more unique National Atmospheric Deposition Program (NADP) sites in a sample year used to build regional mixed effects models of smoke-rain effects on wet deposition.

Table S4. Descriptive statistics of total annual smoke-rain days by season, climate region, and by year.

Table S5. Marginal predictions of expected weekly wet deposition for nine ions across 0–6 smoke-rain days per week. Predictions are derived from gamma regression models with a log link and are evaluated under reference conditions corresponding to summer 2022, with weekly precipitation held at its mean (27.8 mm).

Table S6. Multiplicative effects of smoke-rain days on wet deposition, expressed as exponentiated coefficients (exp (β)), from gamma mixed-effects models. Coefficients represent multiplicative differences in expected weekly ion wet deposition as the number of smoke-rain days within a week increases, holding weekly precipitation amount, season, and year constant and accounting for repeated measurements within sites. Values greater than 1 indicate higher expected deposition in weeks with more smoke-rain days relative to non-smoke-rain days (e.g., exp (β) = 1.30 corresponds to 30% higher expected deposition), whereas values less than 1 indicate lower expected deposition (e.g., exp (β) = 0.88 corresponds to 22% lower expected deposition).

Table S7. Descriptive statistics of total annual smoke days by season, climate region, and by year.

Text S1. Wet deposition attributable to smoke-rain for Ca^{2+}, Mg^{2+}, SO_4^{2-}, NO_3^-, Na^+, Cl^- at National Atmospheric Deposition Program (NADP) sites for the years 2014, 2020, and 2022.

For Ca^{2+}, we estimate that the share of annual wet deposition attributable to smoke-rain days was as much as 20% in 2022 (0.242 kg Ca^{2+}/ha/yr). By comparison, wet deposition from smoke-rain was much lower in 2014 (0.034 kg Ca^{2+}/ha/yr) and 2020 (0.059 kg Ca^{2+}/ha/yr). Smoke-rain-affected wet deposition comprised <5% of annual deposition of Mg^{2+}, NO_3^-, and SO_4^{2-} in 2014 (0.007 kg Mg^{2+}/ha/yr, 0.107 kg NO_3^-/ha/yr, 0.087 kg SO_4^{2-}/ha/yr) and 2020 (0.012 kg Mg^{2+}/ha/yr, 0.111 kg NO_3^-/ha/yr, 0.085 kg SO_4^{2-} /ha/yr) but anywhere from 10-15% in 2022 (0.041 kg Mg^{2+}/ha/yr, 0.427 kg NO_3^-/ha/yr, 0.277 kg SO_4^{2-} /ha/yr). The influence of smoke-rain on annual Na^+ and Cl^- deposition was negligible in all study years (0.004-0.026 kg Na^+/ha/yr, 0.007-0.044 kg Cl^-/ha/yr).

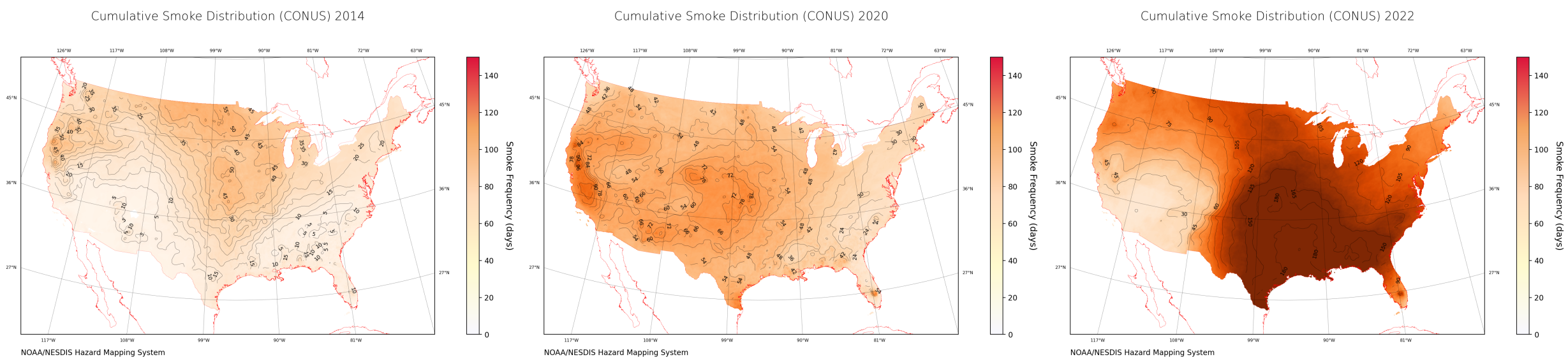

Fig. S1. Cumulative smoke distribution for the contiguous U.S. derived from the Hazard Mapping System (HMS) Fire and Smoke Product. Data show the total number of days when smoke covered the U.S. Contour lines depict spatial variability in the number of observed smoke days. Data accessed February 1, 2026, at https://www.ospo.noaa.gov/Products/land/hms.html

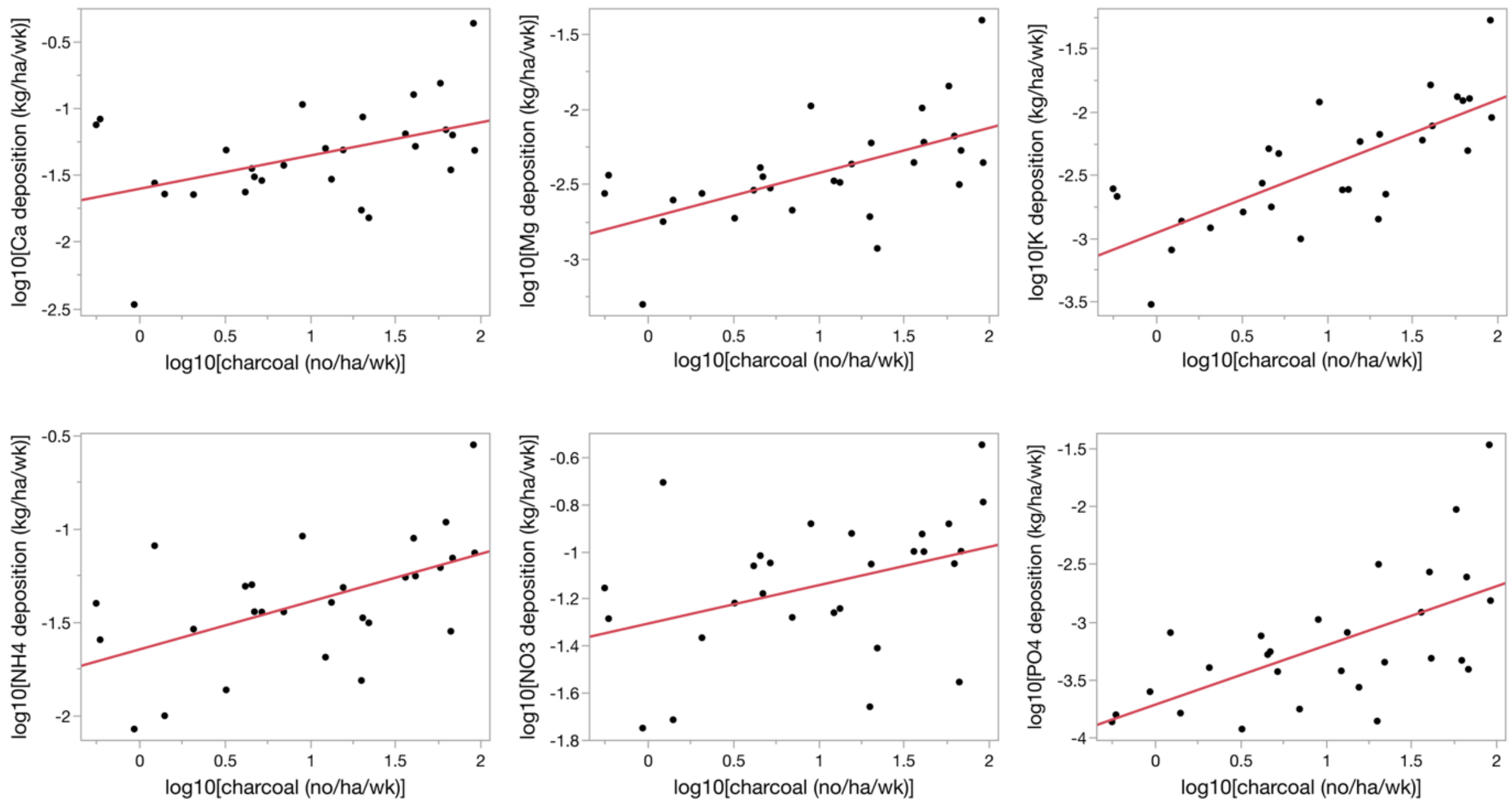

Fig. S2. Significant relationships between nutrient deposition (kg/ha/wk) and charcoal deposition counted on sample filters for 36 filters collected at 5 sites in the western U.S. in summer 2020. Potassium, PO_4^{3-}, and Mg^{2+} deposition exhibited moderate relationships (K^+: $R^2 = 0.56$, $p < 0.0001$; PO_4^{3-}: $R^2 = 0.38$, p = 0.0005; Mg^{2+}: $R^2 = 0.34$, p = 0.0012), while Ca^{2+}, NH_4^+, and NO_3^- exhibited weak relationships (Ca^{2+}: $R^2 = 0.20$, p = 0.0174; NH_4^+: $R^2 = 0.29$, p = 0.0031; NO_3^-: $R^2 = 0.15$, p = 0.0454) with charcoal on the sample filters.

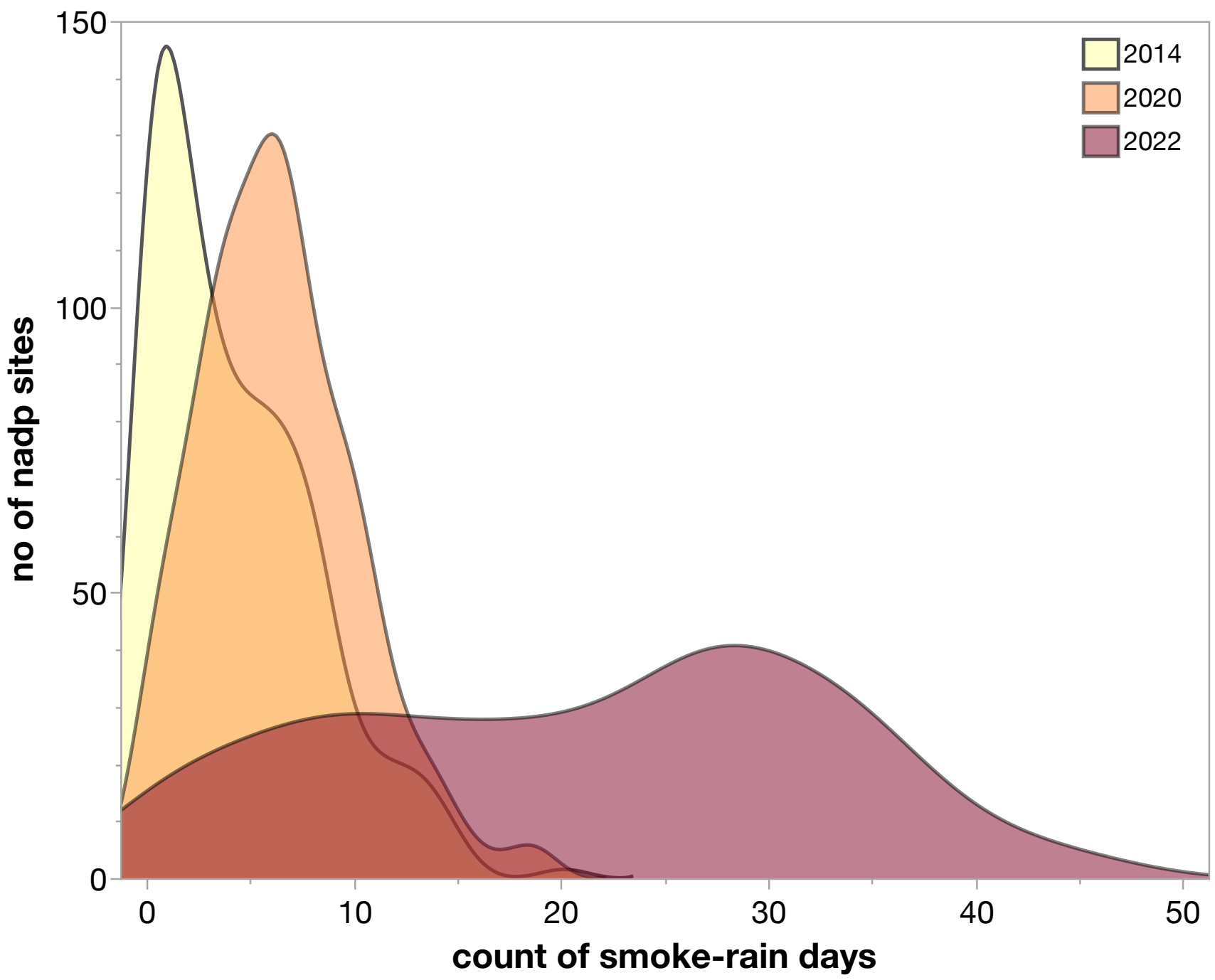

Fig. S3. Distribution of smoke-rain days (i.e., when smoke and rainfall occur at the site on the same day) at National Atmospheric Deposition Program (NADP) sites in 2014, 2020, and 2022.

Table S1. National Atmospheric Deposition Program (NADP) wet deposition samples (*n*=891) included in the dataset and labeled with invalcode "c". Breakdown includes number and percentage of samples in the dataset and broken out by climate region.

Contamination Sources	Number affected	Percent of samples in analytic dataset
Bird Droppings	64	0.3%
Cloudy/Discolored	77	0.3%
Soot/Ash/Dirt	420	1.7%
Insects/Animals	273	1.1%
Plant Material	416	1.7%
Handling	13	0.1%

Climate Region	Number	Bird Droppings	Cloudy/ Discolored	Soot/ Ash/ Dirt	Insects/ Animals	Plant Material	Handling
Alaska & the Arctic	6	0	1	1	0	3	0
Caribbean	0	0	0	0	0	0	0
Northeast	120	18	10	35	42	58	2
Northeastern Forest	5	1	0	2	3	2	0
Northern Rockies	117	2	4	63	22	38	4
Northwest	56	2	5	34	19	27	2
Northwestern Forest	7	0	3	3	2	3	0
Ohio Valley	86	8	8	21	39	43	0
Pacific Coast	2	0	0	1	0	0	0
Prairies	6	0	0	5	2	2	0
South	104	5	6	43	32	48	1
South British Columbia Mountains	3	0	0	2	1	1	0
Southeast	77	17	6	41	42	48	2
Southwest	135	1	22	88	20	56	1
Upper Midwest	136	10	6	55	40	73	1
West	31	0	6	16	9	14	0

Table S2. Criteria used by the National Atmospheric Deposition Program (NADP) and in this study to exclude cases from the analytic file.

Rationale	Exclusion criteria	Cases dropped
Values not comparable	Too few analysis hours (<144)	880
	Too many analysis hours (>194)	868
Invalid samples	Bulk sample and bulk contaminated sample	235
	Bulk sample with major field error and bulk sample with insufficient sample	5
	Contamination (except during smoke-rain events)	2,715
	Major field protocol issue/error or extended field hold time	35
	No sample submitted	67
	Precipitation unknown	184
	Undefined sample	212
	Undefined sample and contaminated	57
	Undefined, insufficient sample	6
	Insufficient sample in bottle	1,464
Missing key data	No smoke-rain data	2,399
	No ion or PH data	5,101
	Insufficient rainfall data (missing rainfall data for ≥25% of observation days)	32

Table S3. Climate regions with 5 or more unique National Atmospheric Deposition Program (NADP) sites in a sample year used to build regional mixed effects models of smoke-rain effects on wet deposition.

Climate Region	Across Years	2014	2020	2022
Alaska & the Arctic	6	5	5	5
Caribbean	3	2	2	3
Northeast	57	47	49	50
Northeastern Forest	1	1	1	1
Northern Rockies	23	21	20	22
Northwest	15	13	12	14
Northwestern Forest	4	1	1	3
Ohio Valley	29	25	26	24
Pacific Coast	1	1	1	1
Prairies	3	1	3	2
South	25	21	22	24
South British Columbia Mountains	2	2	2	2
Southeast	34	31	28	29
Southwest	37	27	33	34
Upper Midwest	29	21	25	27
West	14	14	14	14

Table S4. Descriptive statistics of total annual smoke-rain days by season, climate region, and by year.

	2014					2020					2022				
	N	Mean	Median	Min	Max	N	Mean	Median	Min	Max	N	Mean	Median	Min	Max
**All Sites**	233	4.23	3.50	0.00	20.13	244	6.34	6.13	0.00	18.93	256	20.98	22.15	0.00	47.25
**By Season**															
Winter	232	0.01	0.00	0.00	2.63	242	0.05	0.00	0.00	1.75	255	0.10	0.00	0.00	3.50
Spring	231	0.24	0.00	0.00	2.63	241	0.22	0.00	0.00	12.93	250	4.45	3.50	0.00	21.88
Summer	231	3.77	2.63	0.00	18.38	237	3.49	2.63	0.00	15.43	247	13.72	14.88	0.00	31.50
Fall	232	0.20	0.00	0.00	2.72	237	2.75	2.63	0.00	8.75	249	3.31	2.63	0.00	11.38
**By Climate Region**															
Alaska & the Arctic	5	0.35	0.00	0.00	0.88	5	1.93	1.75	0.88	2.63	5	10.70	10.50	8.00	13.13
Caribbean	2	0.00	0.00	0.00	0.00	2	0.00	0.00	0.00	0.00	3	1.46	0.00	0.00	4.38
Northeast	47	5.09	6.03	0.00	11.38	49	6.06	6.13	0.00	12.25	50	22.51	23.98	8.17	37.58
Northeastern Forest	1	7.00	7.00	7.00	7.00	1	6.13	6.13	6.13	6.13	1	26.25	26.25	26.25	26.25
Northern Rockies	21	6.77	6.28	0.00	13.65	20	7.18	6.42	0.56	17.00	23	18.19	20.13	5.28	27.40
Northwest	13	3.19	2.63	0.00	6.13	12	6.17	6.66	0.00	11.38	14	7.61	8.37	0.00	12.25
Northwestern Forest	1	13.13	13.13	13.13	13.13	1	0.00	0.00	0.00	0.00	3	23.45	23.43	21.17	25.76
Ohio Valley	25	4.88	5.25	0.00	8.88	26	6.65	6.56	1.75	10.50	24	28.79	30.87	2.88	40.25
Pacific Coast	1	0.88	0.88	0.88	0.88	1	3.40	3.40	3.40	3.40	1	7.06	7.06	7.06	7.06
Prairies	1	14.88	14.88	14.88	14.88	3	2.01	1.75	0.00	4.28	2	21.09	21.09	20.03	22.15
South	21	2.81	1.75	0.00	9.63	22	6.76	7.58	1.18	13.13	24	26.18	27.27	0.00	47.25
South British Columbia Mountains	2	0.00	0.00	0.00	0.00	2	2.25	2.25	0.88	3.63	2	5.69	5.69	4.38	7.00
Southeast	31	1.44	0.88	0.00	6.13	28	5.13	4.38	0.88	18.67	29	28.39	28.88	3.53	42.67
Southwest	27	2.66	1.65	0.00	8.75	33	8.91	7.88	0.00	18.93	34	12.19	12.27	0.00	29.05
Upper Midwest	21	10.15	10.50	2.63	20.13	25	8.73	9.63	3.50	15.53	27	33.22	35.00	0.00	44.63
West	14	1.01	0.88	0.00	2.75	14	1.87	1.78	0.00	4.28	14	3.70	3.06	0.00	11.50

Table S5. Marginal predictions of expected weekly wet deposition for nine ions across 0–6 smoke-rain days per week. Predictions are derived from gamma regression models with a log link and are evaluated under reference conditions corresponding to summer 2022, with weekly precipitation held at its mean (27.8 mm).

Smoke-Rain Days	Ca<br>Margin [95% CI]	Mg<br>Margin [95% CI]	K<br>Margin [95% CI]
0	0.036 [0.032, 0.039]	0.006 [0.005, 0.006]	0.007 [0.006, 0.007]
1	0.046 [0.042, 0.051]	0.007 [0.007, 0.008]	0.010 [0.009, 0.010]
2	0.060 [0.054, 0.066]	0.009 [0.009, 0.010]	0.014 [0.012, 0.015]
3	0.079 [0.070, 0.087]	0.012 [0.011, 0.013]	0.019 [0.017, 0.021]
4	0.103 [0.091, 0.115]	0.015 [0.013, 0.017]	0.027 [0.024, 0.030]
5	0.134 [0.117, 0.151]	0.019 [0.017, 0.021]	0.038 [0.033, 0.043]
6	0.175 [0.150, 0.199]	0.024 [0.021, 0.028]	0.053 [0.046, 0.061]
**Smoke-Rain Days**	**Na<br>Margin [95% CI]**	**NH4<br>Margin [95% CI]**	**NO3<br>Margin [95% CI]**
0	0.018 [0.015, 0.021]	0.056 [0.050, 0.061]	0.124 [0.115, 0.132]
1	0.019 [0.016, 0.022]	0.076 [0.068, 0.083]	0.143 [0.134, 0.153]
2	0.020 [0.017, 0.024]	0.103 [0.092, 0.113]	0.166 [0.155, 0.177]
3	0.022 [0.018, 0.025]	0.139 [0.124, 0.154]	0.193 [0.179, 0.207]
4	0.023 [0.019, 0.027]	0.189 [0.167, 0.211]	0.223 [0.205, 0.241]
5	0.024 [0.020, 0.029]	0.257 [0.225, 0.289]	0.259 [0.235, 0.282]
6	0.026 [0.021, 0.031]	0.348 [0.301, 0.396]	0.300 [0.270, 0.330]
**Smoke-Rain Days**	**Cl<br>Margin [95% CI]**	**SO4<br>Margin [95% CI]**	**PO4<br>Margin [95% CI]**
0	0.035 [0.029, 0.040]	0.068 [0.063, 0.073]	0.002 [0.002, 0.002]
1	0.037 [0.031, 0.043]	0.078 [0.073, 0.084]	0.003 [0.003, 0.003]
2	0.039 [0.033, 0.045]	0.090 [0.084, 0.097]	0.004 [0.004, 0.005]
3	0.042 [0.035, 0.048]	0.104 [0.096, 0.113]	0.006 [0.006, 0.007]
4	0.044 [0.037, 0.052]	0.120 [0.110, 0.131]	0.009 [0.008, 0.010]
5	0.047 [0.038, 0.055]	0.139 [0.125, 0.152]	0.013 [0.011, 0.015]
6	0.050 [0.040, 0.059]	0.160 [0.143, 0.178]	0.020 [0.016, 0.023]

Table S6. Multiplicative effects of smoke-rain days on wet deposition, expressed as exponentiated coefficients (exp (β)), from gamma mixed-effects models. Coefficients represent multiplicative differences in expected weekly ion wet deposition as the number of smoke-rain days within a week increases, holding weekly precipitation amount, season, and year constant and accounting for repeated measurements within sites. Values greater than 1 indicate higher expected deposition in weeks with more smoke-rain days relative to non-smoke-rain days (e.g., exp (β) = 1.30 corresponds to 30% higher expected deposition), whereas values less than 1 indicate lower expected deposition (e.g., exp (β) = 0.88 corresponds to 22% lower expected deposition).

	**Ca**	**Mg**	**K**	**Na**	**NO3**	**NH4**	**Cl**	**SO4**	**PO4**
	exp (β) (SE)	exp (β) (SE)	exp (β) (SE)	exp (β) (SE)	exp (β) (SE)	exp (β) (SE)	exp (β) (SE)	exp (β) (SE)	exp (β) (SE)
All sites	1.30 (0.01)**	1.27 (0.01)**	1.41 (0.02)**	1.06 (0.01)**	1.16 (0.01)**	1.36 (0.01)**	1.06 (0.01)**	1.16 (0.01)**	1.46 (0.02)**
Alaska & the Arctic	1.31 (0.11)**	1.30 (0.14)*	1.57 (0.16)**	0.92 (0.12)	1.48 (0.12)**	3.18 (0.35)**	1.12 (0.12)	1.34 (0.12)**	1.03 (0.08)
Northwest	1.70 (0.11)**	1.29 (0.11)**	1.54 (0.12)**	1.27 (0.12)**	1.54 (0.08)**	2.07 (0.13)**	1.08 (0.09)	1.30 (0.07)**	1.28 (0.12)**
Northern Rockies	1.42 (0.06)**	1.44 (0.06)**	1.49 (0.07)**	1.29 (0.06)**	1.35 (0.04)**	1.48 (0.05)**	1.30 (0.05)**	1.30 (0.05)**	1.57 (0.07)**
West	1.98 (0.25)**	1.81 (0.24)**	1.81 (0.25)**	1.44 (0.21)*	1.68 (0.18)**	2.02 (0.25)**	1.36 (0.19)*	1.59 (0.18)**	1.18 (0.15)
Southwest	1.41 (0.05)**	1.39 (0.05)**	1.49 (0.05)**	1.26 (0.05)**	1.20 (0.03)**	1.30 (0.04)**	1.26 (0.04)**	1.24 (0.03)**	1.17 (0.04)**
South	1.28 (0.04)**	1.21 (0.04)**	1.22 (0.05)**	1.06 (0.04)	1.16 (0.03)**	1.19 (0.04)**	1.06 (0.04)	1.08 (0.03)**	1.26 (0.06)**
Upper Midwest	1.26 (0.03)**	1.27 (0.03)**	1.35 (0.04)**	1.03 (0.03)	1.09 (0.02)**	1.19 (0.03)**	1.02 (0.02)	1.12 (0.02)**	1.28 (0.04)**
Ohio Valley	1.24 (0.03)**	1.23 (0.03)**	1.28 (0.04)**	1.03 (0.03)	1.15 (0.02)**	1.26 (0.03)**	1.01 (0.03)	1.12 (0.03)**	1.19 (0.05)**
Southeast	1.20 (0.03)**	1.06 (0.03)*	1.22 (0.04)**	0.88 (0.03)**	1.13 (0.02)**	1.37 (0.04)**	0.87 (0.03)**	1.06 (0.02)**	1.55 (0.08)**
Northeast	1.24 (0.02)**	1.13 (0.03)**	1.41 (0.04)**	0.90 (0.02)**	1.07 (0.02)**	1.34 (0.03)**	0.92 (0.02)**	1.10 (0.02)**	2.04 (0.06)**

Note. Each coefficient comes from a separate gamma-log model. A Bonferroni correction was applied across regional models within each ion to adjust for multiple spatially stratified tests of the same hypothesis. Adjusted p-values are presented for regional models. ** $p<0.01$, * $p<0.05$.

Table S7. Descriptive statistics of total annual smoke days by season, climate region, and by year.

	**2014**					**2020**					**2022**				
	N	Mean	Median	Min	Max	N	Mean	Median	Min	Max	N	Mean	Median	Min	Max
**All Sites**	233	20	19	0	60	244	40	38	0	94	256	89	91	0	193
**By Season**															
Winter	232	0	0	0	4	242	0	0	0	11	255	2	0	0	26
Spring	231	2	1	0	11	241	1	0	0	17	250	19	12	0	78
Summer	231	16	17	0	53	237	16	14	0	41	247	49	50	0	80
Fall	232	2	1	0	17	237	24	24	0	70	249	21	21	0	49
**By Climate Region**															
Alaska & the Arctic	5	4	3	1	9	5	9	10	6	11	5	31	33	23	37
Caribbean	2	0	0	0	0	2	0	0	0	1	3	8	0	0	24
Northeast	47	21	21	13	25	49	27	30	0	36	50	79	79	22	115
Northeastern Forest	1	23	23	23	23	1	28	28	28	28	1	69	69	69	69
Northern Rockies	21	30	25	0	54	20	47	48	3	74	23	87	87	27	155
Northwest	13	27	30	11	38	12	46	48	28	62	14	72	77	0	91
Northwestern Forest	1	55	55	55	55	1	0	0	0	0	3	101	99	97	106
Ohio Valley	25	23	22	7	40	26	38	38	25	49	24	117	124	12	174
Pacific Coast	1	3	3	3	3	1	16	16	16	16	1	27	27	27	27
Prairies	1	60	60	60	60	3	24	34	0	38	2	98	98	94	102
South	21	20	18	3	52	22	52	51	28	75	24	151	166	11	193
South British Columbia Mountains	2	5	5	4	5	2	16	16	16	17	2	37	37	36	38
Southeast	31	9	9	0	17	28	26	26	14	39	29	123	126	37	162
Southwest	27	8	7	1	21	33	60	60	0	81	34	47	48	1	109
Upper Midwest	21	40	41	30	53	25	42	42	32	57	27	103	106	0	150
West	14	18	16	2	47	14	66	69	25	94	14	35	37	12	68
